# Gonorrhœa and Its Effects

**Published:** 1894-06-09

**Authors:** 


### GONORRHŒA AND ITS EFFECTS.

Gonorrhœa is a disease which deserves more careful consideration than is often given to it. As the cause of much pain and of widespread after-consequences, as an illustration of many points in the natural history of infectious disease, and as a severe although indiscriminating moral flagellant, it is full of interest both to the preacher, the pathologist, and the clinician. According to the view one takes of its nature, gonorrhœa may be merely a specific and contagious form of urethritis, or may be a widespread disease affecting eyes, joints, and lymphatics, besides the whole genito-urinary tract from testicles to meatus and from ovaries to vulva; but if we accept the proposition that it is caused by the development of the gonococcus, and the septic organisms which are so often associated with it, we are driven to the wider definition as being alone capable of including all the manifestations of the malady within its net.

As in everything else, the importance attributed to the disease depends largely on the point of view from which it is regarded. There are, doubtless, many very good practitioners who look on gonorrhœa as a simple malady of youth, a sort of sexual measles, not difficult to treat, and more vexing to the spirit than dangerous to the body. It is to be feared, however, that these optimists think lightly of the disease only because the difficult cases pass to the care of other hands. A little experience in certain out-patient departments, or in West-end consulting rooms, those confessionals of this gay city, puts the matter in quite another light, and shows how large a mass of human sorrow and domestic tribulation is traceable to this malady. One must go beyond the gleets, the strictures, and the urinary troubles, recognised on every hand as the sequences of gonorrhœa; the leucorrhœa and failing health from which so many innocent women begin to suffer soon after marriage, the inflamed tubes which make life a torment to them, the resulting sterility, or when that does not occur the inflammations and abscesses arising after parturition, and in the next generation the loss of sight from ophthalmia neonatorum, together with the rickets and tuberculosis resulting from early weaning, these are the things we must look at if we would estimate at all completely the importance of the gonococcus and its doings.

In the treatment of acute suppurating gonorrhœa there is no great difference of opinion as to the propriety of rest, abstinence, alkalies, laxatives, and cleanliness. Everyone admits that the great thing is not to do too much. There is, however, some difference as to local treatment, some medical men maintaining that with the exception of warm bathing no local treatment is admissible; whereas others as strongly maintain the great advantage and the healing efficacy of hot irrigations. It should be understood, however, that at this stage these should be of the mildest nature; they are not to be astringents, but should rather be looked upon as extensions of the general measures for the maintenance of cleanliness; probably very weak solutions of permanganate of potash at a temperature of about 105 degrees are the most useful.

The principal points in dispute relate to the treatment before suppuration is fully established, and after it begins to subside, and to the duration of infectiveness. The question of abortive treatment is one of considerable importance. Some think it not only useless but dangerous in the extreme, others pin their faith upon it. Doubtless it is no use locking the door after the steed is stolen, and the objections to any attempt at abortive treatment where a full discharge has already been set up are obviously valid, but then to call a treatment by such a name, when used at such a time, is an abuse of words. Abortive treatment must be undertaken at the very beginning, in the stage of itching and gumming, not in that of full discharge, and considerable experience seems to have been gathered during recent years, pointing to its efficacy if undertaken at that period. In old days the plan was to inject the anterior portion of the urethra with a solution of nitrate of silver, and trust to luck. In some cases no doubt the result was good, but in others it was so poor that the proceeding gained for itself an ill repute, which it does not easily shake off. Still, it seems to be true that in the earliest stages of the disease the virus lies very superficially, and can be destroyed by prompt therapeusis. Mere injection, however, is hardly sufficient for the purpose. The mucous membrane of the anterior portion of the urethra, and especially of the fossa navicularis, should be thoroughly rubbed, or one may almost say scrubbed, by a pledget of cotton wool wound on a probe, and after this rubbing, after all the mucous and the superficial epithelium has been dislodged from the inflamed membrane, a small quantity of a 2 or 3 per cent. solution of nitrate of silver should be injected and allowed to remain for a few minutes. The injection should be repeated the next day. Of course there is considerable smarting on micturition, and a pretty free discharge for a day or so; but if the disease has not penetrated too deeply it will be aborted, and much suffering will be saved.

It is, however, after the acute stage has subsided that infection is most apt to be spread, for although, under careful treatment, the disease may pass away completely, it happens in a considerable number of cases that some gleety discharge, which is certainly infectious, continues for a long time. In fact, it is very difficult to know when infectivity ceases; it seems very clear that so long as the presence of the gonococcus can be demonstrated the disease is liable to be roused up to full activity by comparatively small causes; but unfortunately the converse is not true, and the absence of the gonococcus is no certain sign of safety. In the great majority of cases by careful living, by the use of balsams, and by local medication to the urethra, in the form of bougies or injections, this gleety condition may be removed. It must be remembered, however, that in a certain number the continuance of this discharge is not due to the persistance of widespread chronic urethritis, but to the existence of small points of ulceration, or patches on which granulations have developed, or to the presence of disease in the posterior part of the urethra continually infecting the parts in front. Against these conditions general medication is helpless, and the use of ordinary lotions is of small avail. In fact, these form the residuum of cases which either go to specialists or drift into the hands of quacks. Not that they are difficult to treat by those who have special experience in the matter, but they require for their cure methods and appliances which are not at everyone's disposal. Sometimes it is an ulcer which requires touching, sometimes granulations on a certain limited portion of the urethra, or perhaps an actual outgrowth may require to be scraped away, or a posterior urethritis may demand local medication. These things do not require extraordinary skill, but they do require special instruments and experience in the use of them; and if with the ordinary treatment which is within the grasp of men engaged in general practice cases of gleet fail to get well, they should, without hesitation, be sent to the specialist, for above all things it is essential that they be cured. They may not, perhaps, be continuously infective, but there can be no doubt that in many cases they are liable to become so under the influence of slight excess, and it is from such cases that the infection of newly-married women, with all its disastrous consequences, most commonly arises, a subject to which we may return in another article.

